# *In Vivo* and *In Vitro* Studies of Th17 Response to Specific Immunotherapy in House Dust Mite-Induced Allergic Rhinitis Patients

**DOI:** 10.1371/journal.pone.0091950

**Published:** 2014-03-19

**Authors:** Chun Wei Li, Han Gui Lu, De Hua Chen, Zhi Bin Lin, De Yun Wang, Tian Ying Li

**Affiliations:** 1 Department of Otolaryngology, National University of Singapore, National University Health System, Singapore, Singapore; 2 Department of Otorhinolaryngology, The First Affiliated Hospital of Sun Yat-Sen University, Guangzhou, China; 3 Department of Otolaryngology-Head and Neck Surgery, Shantou Central Hospital of Sun Yat-sen University, Shantou, China; 4 Department of Allergy, The First Affiliated Hospital of Sun Yat-Sen University, Guangzhou, China; Glaxo Smith Kline, Denmark

## Abstract

T helper (Th)17 cells have been implicated in the development of allergic rhinitis (AR), but their response to specific immunotherapy (SIT) remains unclear. We investigated the impact of SIT on Th17 response and Th1/Th2 changes in AR patients. Blood samples from AR patients (n = 20) who were monosensitized to house dust mite (HDM) were collected before the initiation of SIT (SIT-untreated) and after the end of 2-year SIT (SIT-treated) treatment. Twenty healthy volunteers were recruited as controls. *In vitro* HDM stimulation in peripheral blood mononuclear cells (PBMCs) was also performed. Expression levels of Th17 associated genes were determined in both PBMCs and plasma by PCR and ELISA, while Th17/Th1/Th2/IL10 producing cell proportions were evaluated in PBMCs by flow cytometry. The SIT effect was evaluated by assessing clinical symptoms. mRNA levels of Th17 specific genes (IL17 and RORC) were increased in SIT-untreated AR versus controls, and decreased following SIT treatment. SIT can change the production of Th17 associated genes (reduction of IL17, IL6, and IL23, but increase of IL27) in plasma from AR patients. Th2/Th1 ratio and proportions of Th17 cells were suppressed while IL10 producing CD4^+^ T cells were elevated after SIT. *In vitro* HDM challenge presents concordant patterns with *in vivo* findings: 1) increase of Th2 and Th17 response in AR patients; 2) suppression of IL10 producing CD4^+^ T cells in SIT-untreated AR but elevation in SIT-treated AR patients. Most importantly, a positive correlation between IL17 mRNA/protein levels and clinical symptom scores was observed. SIT significantly inhibits Th17 mediated inflammation in AR and IL17 may be a useful biomarker for both AR severity and SIT therapeutic effect.

**Trial Registration:**

Australian New Zealand Clinical Trials Registry (ANZCTR) ACTRN12613000445774

## Introduction

Allergic rhinitis (AR) is characterized by enhanced T helper (Th)2 cell mediated inflammation, representing the increase of Th2 related cytokines (such as IL4, IL5, and IL13) [1], [2]. The imbalance of the Th1/Th2 reaction was a major pathological phenomenon of AR, but it could not fully explain the mechanism of AR. Recently, more Th subtypes (Regulatory T (Treg) and Th17 cells) have been found to play important roles in allergic diseases [3]. Th17 is considered a separate subset of Th cells, which specifically produces IL17 cytokine and expresses its lineage-specific transcriptional regulators (RORC, retinoic acid receptor-related orphan receptor C) [4]. Th17 cells are involved in autoimmune diseases such as rheumatoid arthritis, multiple sclerosis, and Crohn’s disease [5]. In asthma models, Th17 cells not only enhance neutrophilic, but also eosinophilic infiltration [6]. In addition, large amount of IL17 producing T cells was found in AR patients and IL17 serum levels were related to the clinical severity of AR sensitized with pollen allergens [7], [8]. This evidence suggests that Th17 cells could participate in the pathogenesis of airway allergic inflammation.

In southern China, house dust mite (HDM) is the most common allergen (prevalence rate ranging from 10% to 25%) among AR patients [9]. Currently, allergen specific immunotherapy (SIT) is the only etiological treatment for AR and has a long-term efficacy on improvement of clinical symptoms [10]. In the current study, we sought to investigate the effect of SIT on Th17 cell-mediated inflammatory responses in AR patients who were monosensitized to HDM. Expression of Th17 specific markers (IL17 and RORC) and the proportion of Th cell subtypes (Th17, Th1, Th2, and IL10 producing cells) were determined in peripheral blood mononuclear cells (PBMCs) from AR patients before the initiation of SIT (baseline) and at the end of a 2-year SIT course. These cellular markers were also evaluated in the *in vitro* allergen stimulation PBMC model. Furthermore, the correlation between Th17 gene expression levels and clinical symptoms (assessed by visual analogue scale (VAS)) was evaluated in AR patients who were treated with SIT.

## Materials and Methods

The protocol for this trial and supporting TREND Statement checklist are available as supporting information; see **Checklist S1 and Protocol S1**.

### 2.1 Study Subjects

**Figure 1:**
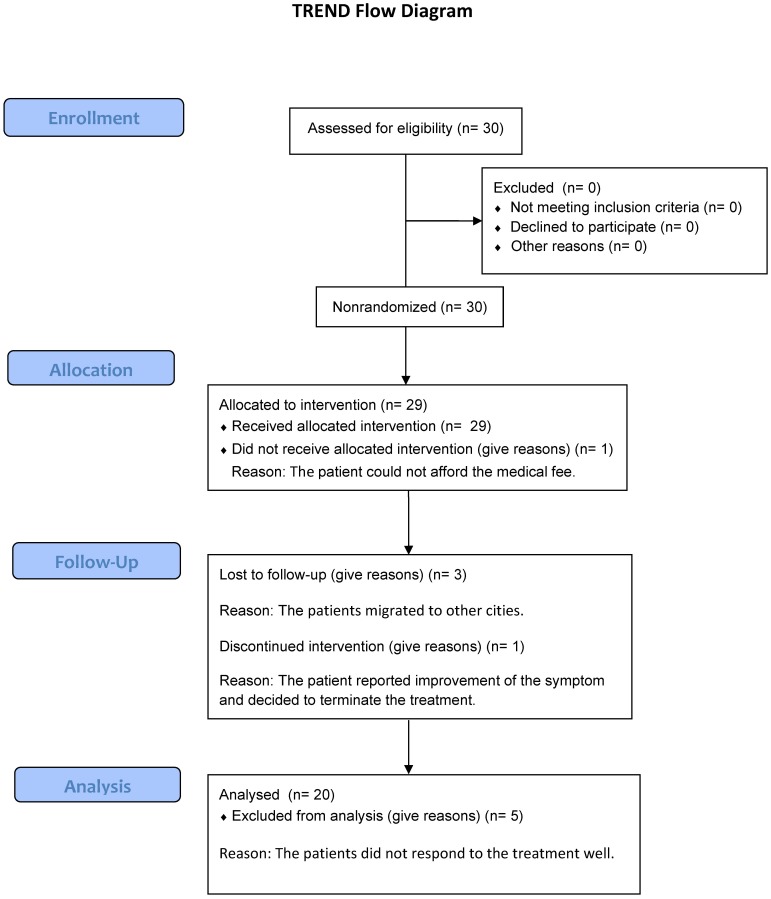
Flow chart of the Transparent Reporting of Evaluations with Nonrandomized Designs (TREND) shows the number of the participants through each stage of the study.

Thirty Chinese patients suffering from persistent AR with significant nasal symptoms (e.g., nasal itching, sneezing, nasal obstruction and rhinorrhea) were recruited in the outpatient clinic in the First Affiliated Medical Hospital of Sun Yat-Sen University from Sept.8^th^, 2008 to Oct.28^th^, 2011 (**Fig. 1**). All AR patients were diagnosed by an ENT/allergy specialist following the criteria from the ARIA document [11]. The inclusion criteria for the study patients were (1) age above 18 years old; (2) having persistent AR symptoms during the past two consecutive years; (3) allergic sensitization to *Dermatophagoides pteronyssinus,* & *Dermatophagoides farinae* confirmed by both skin prick test (SPT) and ImmunoCap (Phadia, Uppsala, Sweden); (4) no previous treatment of SIT; and (5) negative SPT results to other common inhalant allergens (e.g., common pollens, cockroach, fungi, and animal dander).

The serum specific IgE (sIgE) to *Dermatophagoides pteronyssinus* & *Dermatophagoides farinae* was measured by the ImmunoCap test (Phadia) and a value of more than 0.35 kUA/l was considered a positive allergic response. In addition, 20 healthy volunteers (11 males, 9 females, median age 28 years) without any allergy history were selected as a control group. The non-atopic status of healthy controls was double confirmed by a negative SPT to all common inhalant allergens and a negative result of Phadiatop test (Phadia). All recruited subjects (both AR patients and controls) did not have an infection, asthma, autoimmune diseases, or other upper airway diseases such as septal deviation, nasal polyps, or sinusitis before enrolment. Furthermore, the patients and control subjects had not received any form of steroids or SIT at least six months before the study. Both patients and healthy controls were given written consent to undergo the clinical study. The study was approved by the ethics committee of the First Affiliated Medical Hospital of Sun Yat-Sen University in Guangzhou, China.

### 2.2 SIT Protocol

AR patients received subcutaneous SIT for 2 years. SIT was performed by ENT clinicians in the clinic, using with a standardized mite depot-allergen extract (50% *Dermatophagoides pteronyssinus* & and 50% *Dermatophagoides farinae*) according to the recommendations of the manufacturer Allergopharma Joachim Ganzer KG (Reinbek, Germany). The build-up phase (25 weeks) began at 5 TU/ml of the final concentration and increased to 50 TU/ml, 500 TU/ml, and 5000 TU/ml at 5-week intervals as tolerated. Maintenance immunotherapy was administrated with the 5000 TU/ml concentration bi-weekly for 54 weeks and then tri-weekly for 27 weeks (**Fig. S1**). The adjustment in the dosing schedule was mainly based on the tolerance of dose and the occurrence of adverse reactions. None of the study patients have developed any systemic disease (e.g., Asthma) or other nasal diseases (e.g., rhinosinusitis) during the 2-year SIT treatment period. Five patients did not complete the 2-year course of the treatment (**Fig. 1**). The reasons for the loss of follow-up are (1) patients (n = 3) migrated to another city; (2) patient (n = 1) reported improvement of the symptom and decided to terminate the treatment; and (3) patient (n = 1) could not afford the medical fee.

### 2.3 Sample Collection

Blood samples from AR patients were collected before the initiation of SIT (baseline, SIT-untreated) and after the end of the SIT course (the third year, SIT-treated) (**Fig. S1**). Control patients’ blood was also sampled at the beginning of the study. PBMCs (interface) and plasma (upper layer) were isolated from blood samples by using a density centrifugation method with Ficoll-Paque Plus (GE Healthcare Bio-Sciences AB, Uppsala, Sweden). Detail of sample collection procedure was described in the **Material and Methods S1**.

### 2.4 *In vitro* Cell Stimulation

PBMCs were challenged with the HDM allergen. The stimulation protocol was described in the **Material and Methods S1** and a previous study [12].

### 2.5 Quantitative PCR

Total RNA was extracted from PBMCs by using Trizol (Invitrogen) according to the manufacture’s protocol. RNA was reversely transcribed and real-time RT PCR measurement was performed by using ABI 7300 PCR system (Applied Biosystems, Foster City, CA). Relative gene expression was analyzed using the comparative 2^−ΔΔCt^ method as previously described [13]. Sequences of the primer and Tamara probe for IL17, RORC, and glyceraldehyde-3-phosphate dehydrogenase (GAPDH) are shown in the **Material and Methods S1**.

### 2.6 Cytokine Assays

Concentrations of IL6, IL17, IL23 and IL27 in undiluted plasma from SIT-untreated and SIT-treated patients were determined by Enzyme-linked Immunosorbent Assay (ELISA) kits (USCNLIFE, Wuhan, China). IL17 levels were further measured in the supernatants from HDM-stimulated PBMCs. Cytokine production was detected and analyzed by Synergy HT Multi-Mode Microplate Reader (BioTek, Winooski, VT). The details of ELISA analysis are described in the **Material and Methods S1**.

### 2.7 Flow Cytometry

PBMCs were stained with surface markers PE-cy5-labeled anti-human CD4 monoclonal antibody (mAb) and APC-Cy7-labeled anti-human CD3 mAb at room temperature for 30 minutes. The samples were fixed with Fixation Solution (eBioscience, San Diego, CA) at 4°Cfor 20 minutes in dark. Cells were then re-suspended and permeabilized in Permeabilization Buffer (eBioscience) to facilitate the intracellular staining. PBMCs were stained with intracellular cytokines by using PE-labeled anti-human IL4 mAb, PerCP-labeled anti-human IFNG mAb, APC-labeled anti-human IL10 and FITC-labeled anti-human IL17 mAb for 30 minutes at room temperature. Finally, the stained cells were re-suspended in FACS lysing solution (BD Bioscience, San Diego, CA) and stored at 4°C in dark prior to acquisition. All antibodies for both surface and intracellular markers were obtained from BD Bioscience. The PBMC samples stained without primary antibody and stained with species- and subtype-matched isotype controls were served as negative controls. Samples were run on a FACSCalibur Flow Cytometer (BD Biosciences) and were gated by CD3/CD4 positive cells; the data was analyzed by using CellQuest software (BD Biosciences).

### 2.8 VAS Evaluation

The effectiveness of SIT was evaluated based on the patient’s clinical symptoms of AR. The symptom score was analyzed using the VAS system according to the ARIA document [11]. All patients were coded confidentially and VAS was evaluated independently by a clinician in a blinded manner. The fold change of VAS value in AR patients after SIT treatment was calculated by the formula: VAS (SIT-untreated)/VAS (SIT-treated). The details of how to perform VAS evaluation are described in the **Material and Methods S1**.

### 2.9 Statistics

All data were analyzed using the SPSS statistical software V17.0 (SPSS Inc., Chicago, IL). mRNA/protein levels of the cytokines and percentages of the Th cell subtypes, were compared in PBMCs isolated from SIT-untreated, SIT-treated, and control subjects. Wilcoxon matched pairs sign rank test was used to compare the differences of these molecular markers in PBMCs from SIT-treated versus SIT-untreated patients, and HDM-treated versus HDM-untreated *in vitro* samples. The Mann-Whitney two-tailed test was performed to compare the differences of these molecular markers in PBMCs between either SIT-treated or SIT-untreated subjects, versus controls. Change of VAS value was analyzed between SIT-untreated and SIT-treated patients by Wilcoxon matched pairs sign rank test. The correlation between VAS values versus mRNA/protein levels of IL17 and RORC, and VAS fold change versus mRNA/protein level fold change of IL17 and RORC in AR patients were evaluated by Spearman rank analysis. Statistical significance was accepted when the *p*-value <0.05.

## Results

### 3.1 Patient Characters

The demographic information of the AR patients and controls at baseline is summarized in **Table S1**. More than 50% of the AR patients demonstrated highest sensitivity to HDM (i.e., grade 4 for skin prick test or grade 6 for sIgE test) (**Table S1**). VAS was applied to assess the clinical response of patients receiving SIT. The symptoms from most patients (80%, 20/25) were improved after treatment, showing a significant reduction (3.39-fold) of VAS value (**Fig. S2)**. The nasal symptoms from five patients were not relieved following the therapy. These patients were considered non response to SIT and were not included in the molecular data analysis of this study (**Fig. 1**). Therefore, the samples from the twenty patients with significant response to the treatment were used in the following *in vivo* and *in vitro* studies.

### 3.2 *In vivo* Study

mRNA/protein levels of Th17 associated genes and specific cytokines of Th cell subsets were measured in PBMCs from 20 AR patients (before the initiation and after the end of SIT) and 20 healthy controls (**Fig. S1**). **Table S2** summarized the values of all markers in each study group.

#### 3.2.1 Th17 specific gene mRNA expression

**Figure 2:**
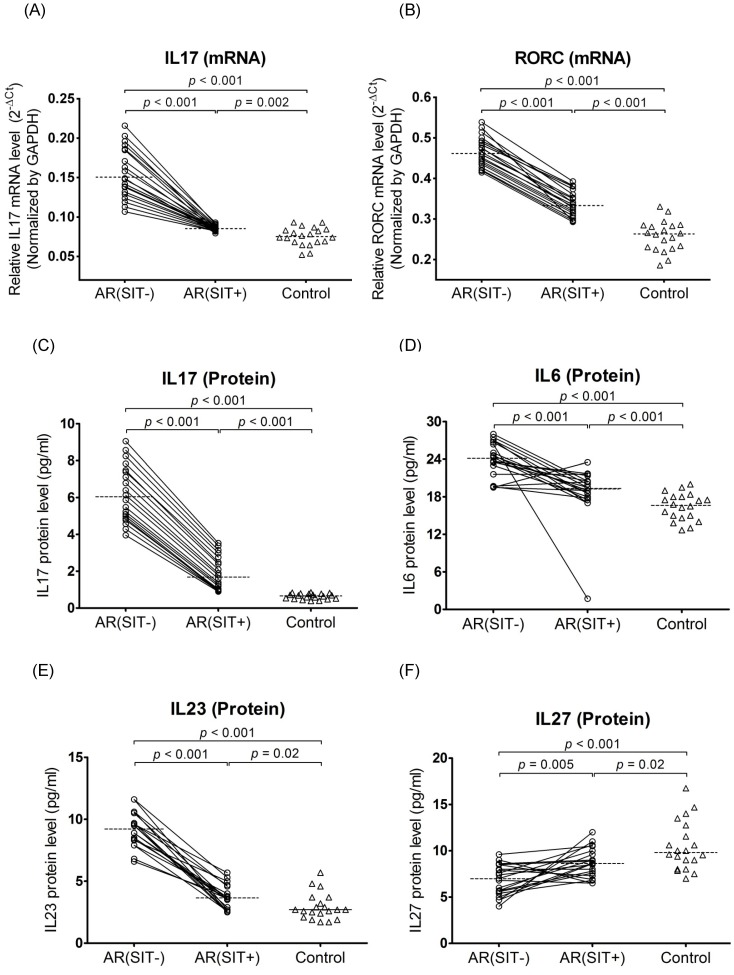
Quantitative determination of mRNA by real-time RT PCR for IL17 (A) and RORC (B) in PBMCs and evaluation of protein production by ELISA for IL17 (C), IL6 (D), IL23 (E), and IL27 (F) in plasma. The samples were collected from AR patients (open circles) and the controls (open triangles). Connecting lines indicates data obtained in the same subject before and after SIT treatment. Medians are shown by horizontal dashed lines. Statistical analyses were performed by Wilcoxon matched pairs sign rank test for paired comparison (SIT-untreated vs. SIT-treated patients); whileMann-Whitney two-tailed test for unpaired comparison (SIT-untreated or SIT-treated vs. control subjects).

Before SIT, mRNA levels of IL17 (2.0-fold) and RORC (1.8-fold) were significantly increased in AR patients compared to controls. After SIT, IL17 (1.8-fold) and RORC (1.4-fold) mRNA expression was significantly down-regulated in AR patients. However, IL17 and RORC were not completely normalized by SIT as the mRNA levels were still higher in AR patients compared to controls (**Fig. 2**).

#### 3.2.2 Th17 associated gene protein expression

Expression levels of plasma Th17 related cytokines (including IL17, IL6, IL23, and IL27) were also assessed in all studied subjects by ELISA. Except for the significant decrease of IL27 (1.4-fold), the protein production of IL17 (9.1-fold), IL6 (1.5-fold), and IL23 (3.4-fold) was significantly increased in plasma from AR patients before SIT compared with those in controls. Moreover, significant up-regulation of IL27 (1.3-fold) and down-regulation of IL17 (3.6-fold), IL6 (1.3-fold), and IL23 (2.4-fold) were found in AR patients after SIT treatment (**Fig. 2**). Similar to the RNA level, there were still significant differences of these proteins’ expression between SIT-treated AR and control subjects (**Fig. 2**).

#### 3.2.3 Proportions of Th cell subsets

**Figure 3:**
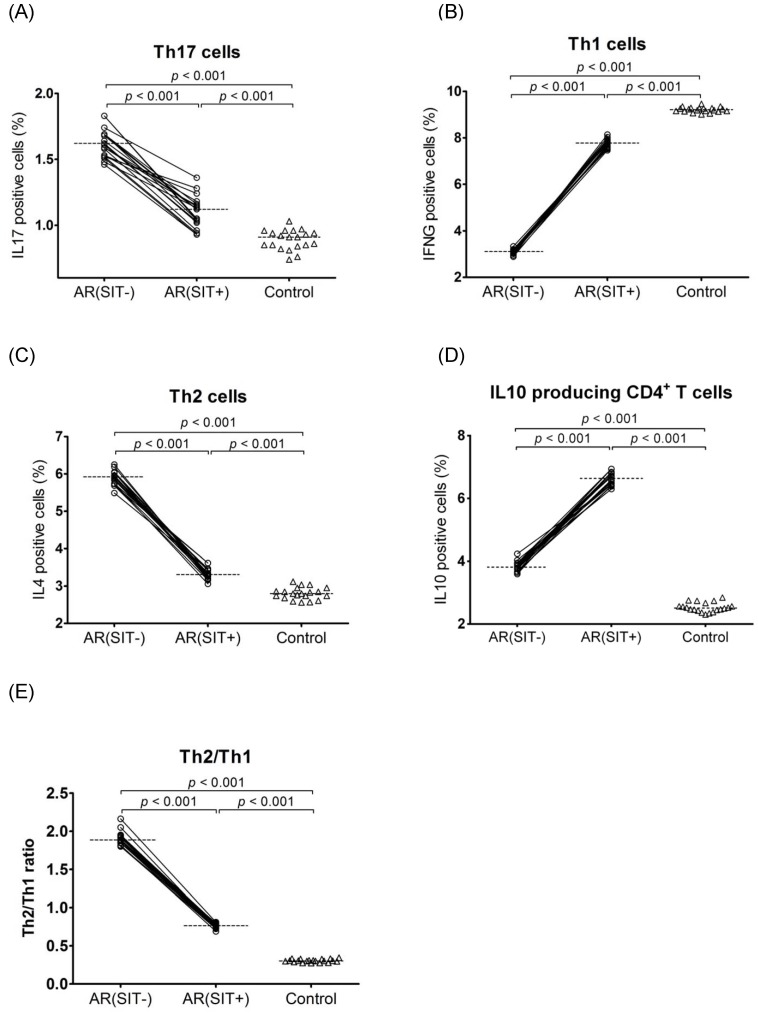
Determination of Th cell subset proportion by determining their specific cytokines IL17 (A), IFNG (B), IL4 (C), and IL10 (D) by flow cytometry in PBMCs. The Th2/Th1 ratio (E) was further calculated. The samples were collected from AR patients (open circles) and the controls (open triangles). Connecting lines indicate data obtained in the same subject before and after SIT treatment. Medians are shown by horizontal dashed lines. Statistical analyses were performed by Wilcoxon matched pairs sign rank test for paired comparison (SIT-untreated vs. SIT-treated patients); while Mann-Whiney two-tailed test for unpaired comparison (SIT-untreated or SIT-treated vs. control subjects).

Evaluation of Th cell subsets were examined by detecting the intracellular IL17 (Th17), IFNG (Th1), IL4 (Th2), and IL10 expression levels in PBMCs from both AR and control subjects. In SIT-untreated AR patients versus controls, there was a significant increase of IL17 (1.60% vs. 0.91%, 1.75-fold), IL4 (5.92% vs. 2.80%, 2.11-fold), and IL10 (3.79% vs. 2.51%, 1.51-fold) expressing cells but a decrease of IFNG (3.11% vs. 9.22%, 2.96-fold) expressing cells (**Fig. 3**). After a 2-year SIT course, the percentages of IL17 (1.60% vs. 1.12%, 1.51-fold) and IL4 (5.92% vs. 3.31%, 1.78-fold) positive cells were significantly reduced while IL10 (3.79% vs. 6.65%, 1.75-fold) and IFNG (3.11% vs. 7.78%, 2.49-fold) positive cells were elevated in SIT-treated AR patients as compared to SIT-untreated patients (**Fig. 3**). In addition, the Th2 (IL4)/Th1 (IFNG) ratio (median = 1.8) was significantly higher in SIT-untreated AR than in controls; but the ratio was reversed (median = 0.7) in SIT-treated AR (**Fig. 3**). Although the response of Th cell subsets to SIT was apparent, there was a significantly higher percentage of Th17, Th2, and IL10 producing CD4^+^ T cells but lower percentage of Th1 cells in SIT-treated AR patients versus controls (**Fig. 3**). Representative pictures of the flow cytometry data are shown in **Fig. S3**.

### 3.3 *In vitro* Study

To mimic the functional response of Th17 and other Th cell subpopulations to HDM allergens, an *in vitro* allergen stimulation study was performed to compare mRNA/protein expression of Th17 specific genes and Th cellular markers in PBMCs from AR patients (both SIT-untreated and SIT-treated) and control subjects (**Fig. S1**). **Table S2** summarized the values of all markers in each study group tested *in vitro*.

#### 3.3.1 Th17 specific gene mRNA/protein expression

**Figure 4:**
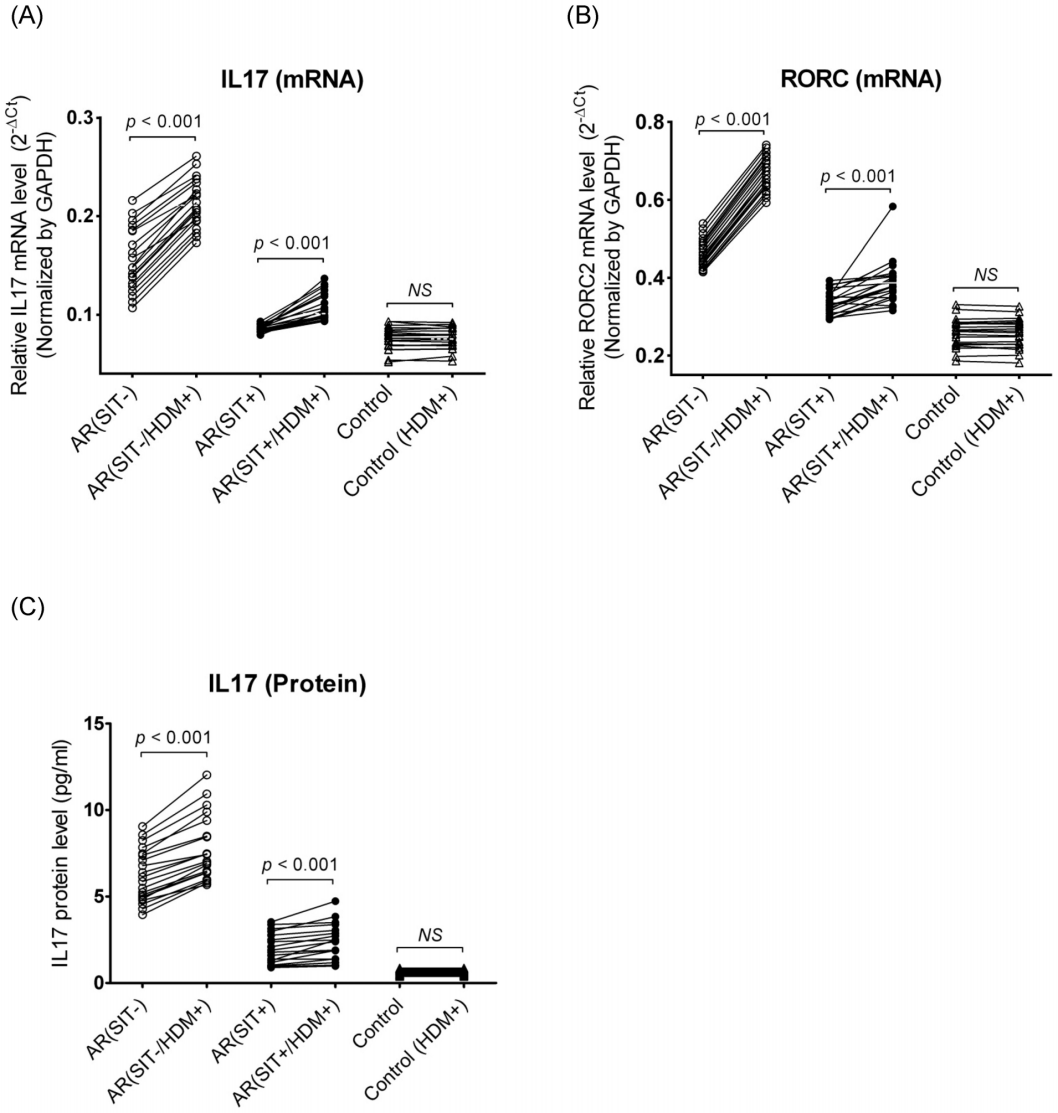
Quantitative determination of mRNA by real-time RT PCR for IL17 (A) and RORC (B) in PBMCs and evaluation of protein production by ELISA for IL17 (C) in supernatant after HDM challenge. The samples were collected from SIT-untreated AR patients (open circles), SIT-treated AR patients (close circles) and the controls (open triangles). Connecting lines indicate data obtained in the same subject before and after HDM stimulation. Statistical analyses were performed by Wilcoxon matched pairs sign rank test. “*NS*” indicates not significantly different.

Following a 3-day *in vitro* stimulation, there were significant increases of mRNA levels of IL17 (1.46-fold) and RORC (1.44-fold) in PBMCs and secreted IL17 protein (1.25-fold) in the supernatant from SIT-untreated AR patients (**Fig. 4**). The fold changes of these Th17 related genes (IL17 mRNA, 1.17-fold; RORC mRNA, 1.15-fold; IL17 protein, 1.12-fold) were smaller in SIT-treated AR subjects after challenge compared to the SIT-untreated AR subjects, but reached a significant difference (**Fig. 4**). However, no significant response of these markers to HDM stimulation was found in controls.

#### 3.3.2 Proprotions of Th cell subsets

**Figure 5:**
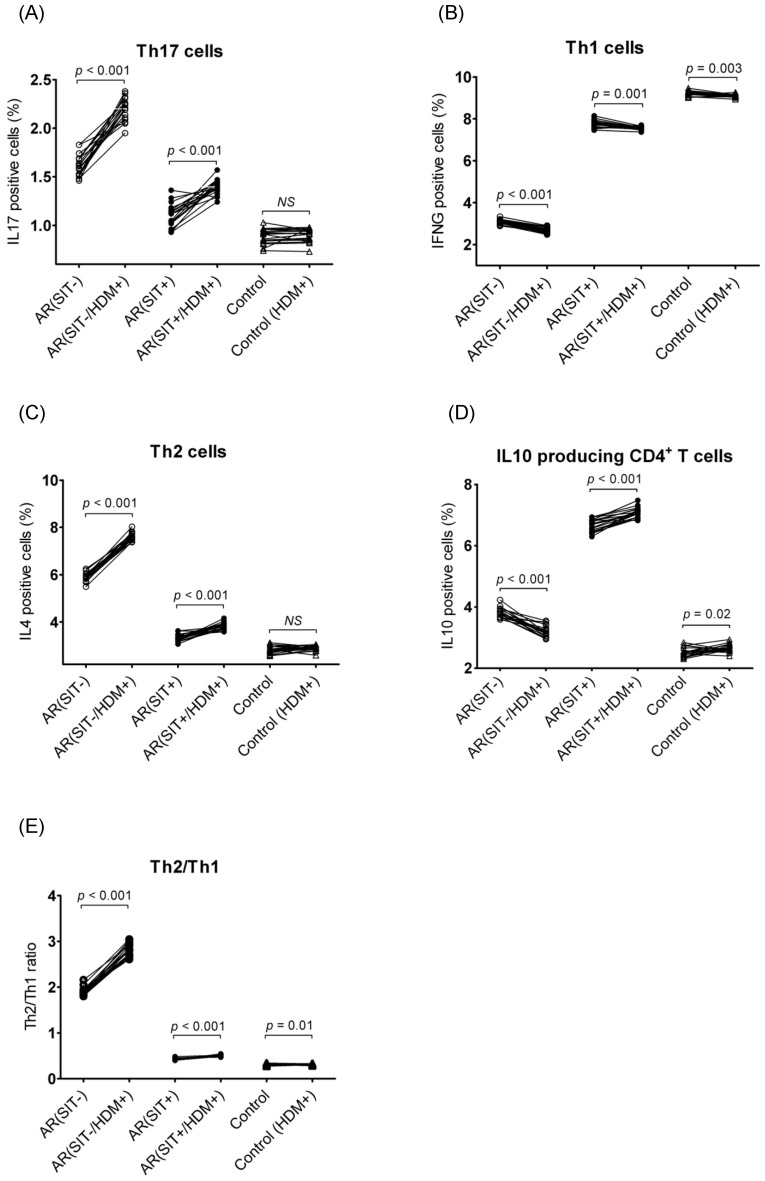
Determination of Th cell subset proportion by determining their specific cytokines IL17 (A), IFNG (B), IL4 (C), and IL10 (D) by flow cytometry in PBMCs after HDM challenge. The samples were collected from SIT-untreated AR patients (open circles), SIT-treated AR patients (close circles) and the controls (open triangles). Connecting lines indicate data obtained in the same subject before and after HDM stimulation. Statistical analyses were performed by Wilcoxon matched pairs sign rank test. “*NS*” indicates not significantly different.

Challenge with allergens in PBMCs resulted in up-regulation of Th17 (IL17, 1.36-fold) and Th2 (IL4, 1.30-fold) specific cytokines and the Th2/Th1 ratio (1.47-fold) but down-regulation of the Th1 (IFNG, 1.14-fold) and IL10 producing (IL10, 1.21-fold) cell cytokines in AR patients before SIT treatment (**Fig. 5**). In SIT-treated subjects, these cytokines showed less change after stimulation as compared to the patients without SIT therapy, although the difference was significant: increase of IL17 (1.27-fold), IL4 (1.15-fold), Th2/Th1 (1.16-fold), and IL10 (1.06-fold), while there was a decrease of IFNG (1.03-fold) (**Fig. 5**). Regarding the controls, the differences of Th1 (1.02-fold down-regulation) and IL10 producing CD4^+^ T cells’ (1.06-fold up-regulation) markers reached a statistical significance after stimulation but were minor compared to the changes found in AR patients. (**Fig. 5**).

### 3.4 Relationship between mRNA/Protein Expression Levels of Th17 Specific Genes and Clinical Symptoms

**Figure 6:**
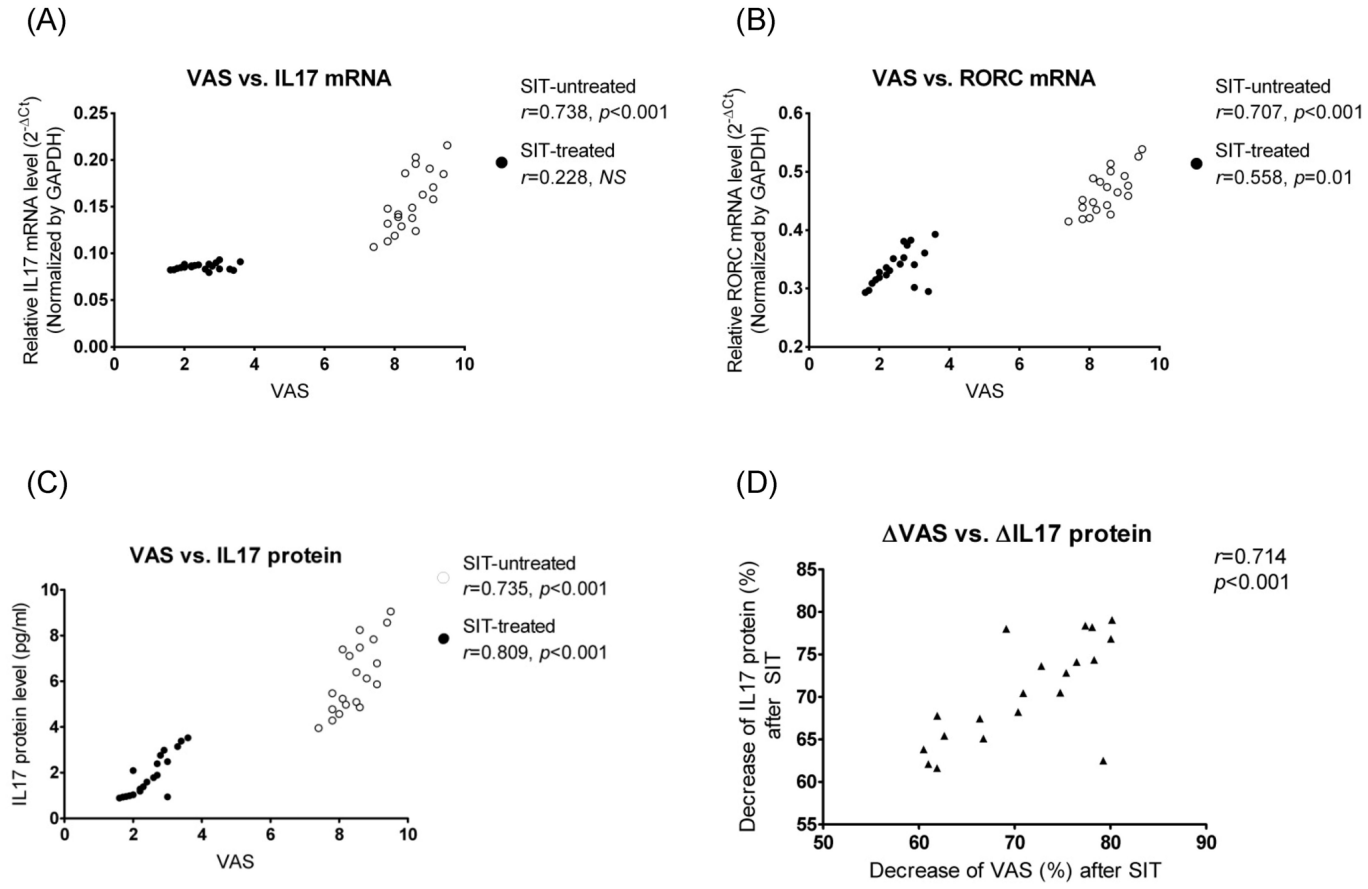
Relationship between VAS versus IL17 protein (A), IL17 mRNA (B), and RORC mRNA (C), and between changes of VAS versus changes of plasma IL17 protein (D) were also illustrated. ΔVAS referred to the ratio of (VAS_SIT−_−VAS_SIT+_)/VAS_SIT−_, and ΔIL17 referred to the ratio (in percentage) of (IL17_SIT−_−IL17_SIT+_)/IL17_SIT−_; Spearman correlation analysis was performed. “*NS*” indicates not significantly different.

In AR patients before SIT, mRNA levels of IL17 (*r* = 0.738, *p*<0.001) and RORC (*r* = 0.707, *p*<0.001) in PBMCs and plasma IL17 protein (*r* = 0.735, *p*<0.001) level had a significant positive correlation with the symptom severity (**Fig. 6**). At the end of SIT, RORC (*r* = 0.558, *p* = 0.01) mRNA and IL17 (*r* = 0.809, *p*<0.001) protein levels were positively correlated with VAS values (**Fig. 6**). We further analyzed the relationship between fold changes of plasma IL17 concentration and fold changes of VAS in SIT-treated patients, and found that the decrease of IL17 levels were positively correlated with the decrease of VAS scores after SIT treatment (**Fig. 6**).

## Discussion

Th17 cell has been found to play important roles in both neutrophil and eosinophil mediated inflammation in asthma [14], [15], but its function in AR and its response to SIT have been studied less. In the current study, we demonstrated that the elevated mRNA and protein levels of IL17 in HDM monosensitized AR patients was related to the clinical symptom severity, and for the first time we showed those Th17 associated genes can be significantly inhibited by subcutaneous SIT in AR. Other Th cell subpopulations were also changed after SIT treatment. *In vitro* allergen stimulation enhanced Th2 and Th17 activities in PBMCs isolated from AR patients, which was concordant to the *in vivo* situation.

Th17 cells mediate airway inflammation by producing its unique cytokine IL17 to induce those proinflammatory genes from both structure cells (epithelial cells and fibroblasts) and immune cells (macrophages and dendritic cells) [3]. Regulation of Th17 cell differentiation is tightly controlled by transcription factors and cytokines [4]. RORC is considered a candidate master directing the Th17 cell lineage differentiation [16]. IL6 and IL23 not only mediate the survival and development of Th17 cells, but also induce constitutive IL17 expression [17]. In contrast, IL27 inhibits Th17 generation and those gene molecules associated with Th17 function [18]. The results showed that the increase of RORC, IL6 and IL23, but decrease of IL27 may indicate the enhanced Th17 immune activity in HDM-induced AR.

SIT has been considered the only treatment that interferes with the basic pathological mechanism of the allergic disease (such as AR and asthma) [19]. In line with the previous studies, we also found SIT was effective in relieving AR symptoms after a 2-year therapy course [19]. This is the first *in vivo* study to present the response of IL17 and its associated genes to SIT in HDM-induced AR to date. Our data showed that SIT could significantly suppress expression of IL17, RORC, IL6 and IL23, but up-regulate IL27 (an inhibitor of IL17) levels in AR, indicating the alleviation of Th17-mediated inflammation by SIT intervention. More importantly, IL17 and RORC expression levels correlated well to the symptom scores in AR patients (both SIT-untreated and SIT-treated). Similar data was also reported by Ciprandi et al. demonstrating a down-regulation of IL17 protein levels in pollen-sensitized AR patients (Caucasians) after sublingual immunotherapy [20]. These results suggest that IL17 may be a useful marker for AR symptom severity and even an indicator for the therapeutic efficacy.

Flow cytometry results demonstrated that Th2-mediated inflammation was dominant in SIT-untreated AR subjects as a significantly high Th2/Th1 ratio (median = 1.8) was observed. A shift of Th2 towards Th1 (median of Th2/Th1 ratio = 0.7) was found in PBMCs from SIT-treated patients, indicating the Th2 response was blunted. In addition, the IL17 positive cells were reduced in PBMCs from AR patients following the SIT treatment. The results imply that besides predominant Th2 immunity, abnormal Th17 activity is also involved in AR. We also detected a higher percentage of IL10 producing CD4^+^ T cells in AR before SIT versus controls, and the IL10 positive cells was further elevated after SIT. However, because there was a lack of FOXP3 detection, the IL10^+^ cells here could not be considered Treg cells. Nevertheless, our data are in agreement with previous reports showing that SIT was able to trigger Th2 to Th1 switching, paralleled with the increased proportion of IL10 producing cells [21]–[23]. Moreover, IL10 producing Treg cells can suppress Th17 response during inflammation [24]. These findings suggest the induction of IL10 in T cells during chronic allergic reactions (e.g., SIT-untreated AR) could not effectively curtail the Th2 and Th17 mediated inflammation, but it could be enhanced following SIT in the relieved stage of inflammation (e.g., SIT-treated AR), indicating the response of immunoregulation after SIT therapy.

We further performed the *in vitro* allergen challenge in PBMCs to evaluate the Th17 cell associated genes as well as the responses of Th subsets. Following the stimulation, mRNA of IL17/RORC, protein secretion of IL17, and intracellular IL17 levels were up-regulated in PBMCs from AR patients but not in control subjects; while the increase of Th2 but decrease of Th1 cells was also determined in all AR’s PBMCs. These results show a concordance between *in vivo* and *in vitro* findings, confirming that not only Th2, but also Th17 cells play an important role in the context of allergic inflammation. IL10 producing CD4^+^ T cells showed a distinct response to HDM in PBMCs among different subject groups: reduction of IL10^+^ cells in SIT-untreated AR, but an increase in SIT-treated and control subjects. These different reactions indicate the regulatory function of IL10^+^ cells may be impaired in severe inflammation, while it can be activated in mild inflammatory or healthy status, showing a similar pattern as those found in the *in vivo* situation.

Although the clinical and molecular improvement was obvious in AR subjects who received SIT, the levels of IL17 as well as its related markers and the Th cell subsets show a significant difference between SIT-treated AR and controls. Moreover, allergen stimulation was able to promote the Th17 and Th2 inflammatory response in PBMCs from SIT-treated AR patients, although the change of these cytokine expressions was less than that in SIT-untreated AR. This evidence indicates the immunologic changes may not be completely normalized by a 2-year of SIT in AR. As recommended by the literature, a 3-year or longer SIT period may result in consistent long-lasting effects after the cessation of treatment [19].

Some limitations of the current study need to be considered. After excluding the non-compliant patients and the SIT unresponsive patients, only a small number of subjects were analyzed in this study. Follow-up studies are needed in a large number of patients to verify the Th17 response between SIT responsive and unresponsive groups. Another shortcoming is the lack of controlled placebo group. For ethical reasons, it was not reasonable to have AR patients not treated with any form of pharmacologic agents for 2 years. However, we have compared the change of molecular response in each patient before and after treatment, which could be regarded as a self-control design to minimize the placebo effect. In addition, a lack of the evidences of local nasal response (e.g., testing the inflammatory markers in nasal mucosa or nasal lavage) is also a limitation. We could not confirm the impact of systemic changes of T-cell phenotype to the nasal mucosal inflammation following SIT therapy.

In conclusion, our *in vivo* and *in vitro* studies presented the evidence that not only Th2 but also Th17 mediated inflammation was involved in the AR pathological mechanism. The Th17 response was reduced in AR following SIT. The relationship between IL10 producing CD4^+^ T cell and Th17 immunity in AR and its response to SIT needs to be further clarified. The clinical relevance between Th17 and AR suggest that its specific gene, IL17 can be considered a potential biomarker not only for the severity of allergic symptom but also for the therapeutic effect of SIT in AR.

## Supporting Information

Figure S1 **Flow chart of the study showing the experimental design and SIT protocol.** mRNA levels of the genes and proportion of Th cell subsets were evaluated from PBMC samples; while protein production was tested from plasma and cell culture supernatant. Clinical symptom scores were evaluated using the VAS system. AR patients were treated with SIT for 2 years.
(EPS)

Figure S2 **Change of VAS (A) scores before SIT and after SIT in AR patients.** Connecting lines indicate data obtained in the same subject. Statistical analyses were performed by Wilcoxon matched pairs sign rank test. Medians are indicated by horizontal dashed lines.
(EPS)

Figure S3 Representative flow cytometry results show the proportion of Th17, Th1, Th2 and IL10^+^ CD4^+^ T cell subsets in PBMCs from SIT-untreated AR (panel A), SIT-untreated AR with *in vitro* HDM stimulation (panel C), SIT-treated AR (panel E), SIT-treated AR with *in vitro* HDM stimulation (panel G), control subjects (panel I), and control subjects with *in vitro* HDM stimulation (panel K). Panels B, D, F, H, J, L are the pictures for corresponding negative controls. Y-axis describes percentage of IL17 positive cells. X-axis describes percentage of IFN-γ, IL4, and IL10 positive cells, respectively. The cells were gated on CD3/CD4 positive cells.
(EPS)

Table S1 **Characteristics of AR patients and controls at baseline.**
(DOCX)

Table S2 **Summary of the expression levels of IL17- related markers and the percentages of Th17/Th1/Th2/−IL10^+^ CD4^+^ T cell subtypes in different sample types.**
(DOCX)

Checklist S1 **TREND checklist.**
(DOCX)

Material and Methods S1 (DOCX)

Protocol S1 Trial Protocol (DOCX)
