# Supplementary material for: In Vivo and In Vitro Studies of Th17 Response to Specific Immunotherapy in House Dust Mite-Induced Allergic Rhinitis Patients
Source: PLoS One. 2014 Mar 19;9(3):e91950. doi: 10.1371/journal.pone.0091950 (PMC3960160; doi:10.1371/journal.pone.0091950)
Supplement: Table S1 — Characteristics of AR patients and controls at baseline. (DOCX) [file pone.0091950.s004.docx]

**Table S1** Characteristics of AR patients and controls at baseline

|  | AR patients | Controls |
| --- | --- | --- |
| Number of Subjects | 25 | 20 |
| Age (yr) (median) | 28 | 28.5 |
| Gender (Male/Female) | 15/10 | 11/9 |
| SPT (wheal diameter)^*^   - Negative (<3 mm) - Grade 3 (10-15 mm) - Grade 4 (>15 mm) | 0  10  15 | 20  0  0 |
| HDM sIgE^#^   - Grade 4 (17.5-50 KU/L) - Grade 5 (50-100 KU/L) - Grade 6 (>100 KU/L)   Phadiatop   - Negative (<0.35 KU/L) - Positive (>0.35 KU/L) | 3  9  13  0  25 | NA  NA  NA  20  0 |

NA, not applicable;

^*^A wheal diameter greater than 3 mm of the negative saline control was considered SPT positive.

^#^ The serum sIgE to HDM (*Der p* and *Der f*) was measured by the ImmunoCap test and a value of more than 0.35 kUA/l (Grade 1) was considered a positive response.
