# Supplementary material for: In Vivo and In Vitro Studies of Th17 Response to Specific Immunotherapy in House Dust Mite-Induced Allergic Rhinitis Patients
Source: PLoS One. 2014 Mar 19;9(3):e91950. doi: 10.1371/journal.pone.0091950 (PMC3960160; doi:10.1371/journal.pone.0091950)
Supplement: Table S2 — Summary of the expression levels of IL17- related markers and the percentages of Th17/Th1/Th2/−IL10+ CD4+ T cell subtypes in different sample types. (DOCX) [file pone.0091950.s005.docx]

**Table S2** Summary of the expression levels of IL17- related markers and the percentages of Th17/Th1/Th2/–IL10^+^ CD4^+^ T cell subtypes in different sample types

| **Markers** | **Sample types** | ***In vivo* test** | | | ***In vitro* test** | | |
| --- | --- | --- | --- | --- | --- | --- | --- |
|  |  | **AR (SIT-)**  **(median)** | **AR (SIT+)**  **(median)** | **Control**  **(median)** | **AR (SIT-/**  **HDM+)**  **(median)** | **AR (SIT+/**  **HDM+)**  **(median)** | **Control (HDM+)**  **(median)** |
| RORC mRNA | PBMC | 0.462 | 0.334 | 0.263 | 0.669 | 0.378 | 0.261 |
| IL17 mRNA | PBMC | 0.148 | 0.085 | 0.075 | 0.211 | 0.101 | 0.076 |
| IL17 protein (pg/ml) | Plasma/Supernatant | 6.003 | 1.692 | 0.660 | 7.233 | 2.142 | 0.655 |
| IL6 protein (pg/ml) | Plasma | 23.7 | 21.4 | 16.65 | NA | NA | NA |
| IL23 protein (pg/ml) | Plasma | 9.20 | 3.65 | 2.70 | NA | NA | NA |
| IL27 protein (pg/ml) | Plasma | 6.90 | 8.65 | 9.80 | NA | NA | NA |
| IL17+ cells (%) | PBMC | 1.60 | 1.12 | 0.91 | 2.23 | 1.39 | 0.92 |
| IFNG+ cells (%) | PBMC | 3.11 | 7.77 | 9.22 | 2.71 | 7.58 | 9.10 |
| IL4+ cells (%) | PBMC | 5.92 | 3.31 | 2.80 | 7.59 | 3.82 | 2.92 |
| IL10+ cells (%) | PBMC | 3.79 | 6.64 | 2.52 | 3.16 | 7.08 | 2.63 |

^­^NA, Not applicable
