# Supplementary material for: In Vivo and In Vitro Studies of Th17 Response to Specific Immunotherapy in House Dust Mite-Induced Allergic Rhinitis Patients
Source: PLoS One. 2014 Mar 19;9(3):e91950. doi: 10.1371/journal.pone.0091950 (PMC3960160; doi:10.1371/journal.pone.0091950)
Supplement: Material and Methods S1 — (DOCX) [file pone.0091950.s007.docx]

**Materials and Methods S1**

**Sample collection**

The sample collection and procession were carried in a standardized procedure: (1). Two tubes (5 ml each) of whole blood were collected from each subject; (2). Blood samples were coded confidentially, stored in ice and sent to the service lab within 3 hours; (3). One tube was for separation of plasma, and the other was for isolation of PBMC; (4). Freshly isolated PBMC was stimulated with house dust mite (HDM) extracts; (5). HDM un-stimulated and stimulated PBMC were separated into two portions, one for RNA extraction and the other for flow cytometry; (6). Plasma, and cell culture supernatants were stored in small aliquots at -80℃ until use; (7). PBMC pellets were preserved in RNAlater® (Life Technologies) at -80℃, when finishing sample collection, RNAs were isolated and the aliquots were stored at -80℃; (8). A portion of cells were fixed and were performed flow cytomtery experiment immediately. All ELISA and PCR experiments were performed at the same time in the end of sample collection. The sample process and experiments of quantitative PCR, ELISA, and flow cytometry were done by an officially registered commercial service lab (Biolink, Guangzhou, China; website, http://www.biolinkchina.com/).

**Quantitative PCR**

Primer and Tamara probe sequences of IL17, RORC, and GAPDH were designed by using Primer Exress 2.0 (Applied Biosystems) and the sequences were as follows:

IL17 (NCBI Reference Sequence: NM_002190),

Forward Primer: 5’-GGGCCTGGCTTCTGTCTGA-3’;

Reverse Primer: 5’-AAGTTCGTTCTGCCCCATCA-3’;

Probe: 5’-FAM-CAAGGCACCACACAACCCAGAAAGGAG-TAMRA-3’

RORC2 (NCBI Reference Sequence: NM_001001523.1),

Forward Primer: 5’-GGCTTTAAGTTATTTATGTA-3’;

Reverse Primer: 5’-CCTGTATTTATTTGAGCTATT-3’;

Probe: 5’-FAM-CCTACTTTATTTTGTTTGTC-TAMRA-3’

GAPDH (NCBI Reference Sequence: NM_002046.3),

Forward Primer: 5’-CCACTCCTCCACCTTTGACG-3’;

Reverse Primer: 5’-CCAGCAAGAGCACAAGAGGAA-3’;

Probe: 5’-FAM-TGGCATTGCCCTCAACGACCACTTT-TAMRA-3’;

***In vitro* cell stimulation**

PBMCs were cultured with condition medium containing RPMI-1640 (Sigma, St.Louis, MO), 10% heat-inactived fetal bovine serum (Sigma), and 100 U/ml penicillin and 100 μg/ml streptomycin (Invitrogen, Carlsbad, CA). PBMCs were stimulated with HDM allergen for 72h at the optimized concentration (50 μg/ml) [[1](#_ENREF_1)]. Allergen mixture is a standardized mite depot-allergoid (50% *Dermatophagoides pteronyssinus* and 50% *Dermatophagoides farinae*) (Allergopharma, Rheinbek, Germany). At the end of the stimulation, monensin (eBioscience, San Diego, CA) (2 µmol/L), PMA (eBioscience) (20 µg/L) and ionomycin (eBioscience) (1 mg/L) were added to both HDM treated and HDM untreated cultures for 5h to enhance the expression of the cytokines.

**Cytokine assay analysis**

Analysis of the ELISA results was performed under the manufacture’s instruction. Averaged the duplicate readings (optical density, OD) for each standard, control, and sample and then subtracted the average zero standard optical density. Standard curve was created by using commercial software Curveexpert (Hyams.D.G., http://www.curveexpert.net): (1) plotting the mean OD for each standard on the y-axis against the concentration on the x-axis; (2) drawing the curve line with quadratic fit (y=a+bx+cx^2^) regression method. The calculation of cytokine concentration was also done by using Curveexpert. The lower detection limit of IL17, IL6, IL23, and IL27 assays were 0.5 pg/ml, 5 pg/ml, 1.25 pg/ml, and 2.5 pg/ml, respectively.

**VAS evaluation**

The symptom score was analyzed by VAS system according to the ARIA document [[2](#_ENREF_2)]. The recommended method of assessing severity of symptoms (e.g., itching, sneezing, congestion and discharge) is with the use of a VAS recorded by the patient on a 10cm line giving a score on a measurable continuum of 1 to 10. Then subjects were instructed that “Score 0” meant “overall symptoms not all bothersome” and that “Score 10” meant “overall symptoms extremely bothersome”. All patients were coded confidentially and VAS was evaluated both before and after (2 year) SIT independently by a clinician in a blinded manner.

**Reference**

1. Qu SH, Li TY, Ou ZY, Lin ZB, Chen YQ, et al. (2005) Expression of T-bet and its relation with IgE and eosinophil cationic protein in patients with allergic rhinitis. Zhonghua Er Bi Yan Hou Tou Jing Wai Ke Za Zhi 40: 908-911.

2. Bousquet J, Khaltaev N, Cruz AA, Denburg J, Fokkens WJ, et al. (2008) Allergic Rhinitis and its Impact on Asthma (ARIA) 2008 update (in collaboration with the World Health Organization, GA(2)LEN and AllerGen). Allergy 63: 8-160.
