# Supplementary material for: In Vivo and In Vitro Studies of Th17 Response to Specific Immunotherapy in House Dust Mite-Induced Allergic Rhinitis Patients
Source: PLoS One. 2014 Mar 19;9(3):e91950. doi: 10.1371/journal.pone.0091950 (PMC3960160; doi:10.1371/journal.pone.0091950)
Supplement: Protocol S1 Trial Protocol — (DOCX) [file pone.0091950.s008.docx]

**Project title: The molecular mechanism underlying specific immunotherapy in patients with allergic rhinitis**

**Project period：**2008.7—2011.10

**Application department：**Otolaryngology

**Principal investigator：**LI Tian Ying

**Address：**Zhong Shan 2^nd^ road 58, Guang Zhou

**Post code：**510080

**Tel：**87332200-8483

**Email：**litianying@yeah.net

**Application date:** 2008.4.20

**Study background and significance**

**Background**

Allergic rhinitis (AR) is a multi-factorial disease which is influenced by both genetic and environmental factors. The molecular changes underlying AR pathogenesis is characterized by enhanced T helper (Th)2 cell mediated inflammation, representing the increase of Th2 related cytokines (such as IL4, IL5, and IL13). The imbalance of the Th1/Th2 reaction was a major pathological phenomenon of AR, but it could not fully explain the mechanism of AR.

House dust mite (HDM) is also the most prevalent allergen in patients (either adults or children) with AR and/or asthma, especially those stayed in the southern area. The management of AR formulated by ARIA involves education of the patient and the patient’s care giver, allergen avoidance, pharmacotherapy, and specific immunotherapy (SIT). Different from other pharmacological treatment, SIT is considered to be capable of modifying the natural course of AR and its efficacy has been confirmed in treating inhalant allergy caused by pollen, HDM, fungi, and animal allergens. However the mechanisms of SIT in modifying the immune response in AR remain unclear.

**Aims and significance**

In the current study, we sought to investigate the effect of SIT on AR patients who were monosensitized to HDM and the molecular mechanism underlying the AR. The results will be helpful to identify some candidate genes which could represent the AR severity and the SIT clinical efficacy, and further provide a better treatment strategy of SIT.

**Funding source**

This study was supported by grant from Science and Technology Planning Project of Guangdong Province, China (No. 99M04901G).

**Principal investigators (PI) and researchers**

**PI:** Prof. LI Tian Ying, Department of Otorhinolaryngology, The First Affiliated Medical Hospital of Sun Yat-Sen University, Guangzhou, China.

**Co-PI:** Prof. WANG De Yun, Department of Otolaryngology, National University of Singapore, National University Health System, Singapore

Researchers:

**Research scientist:** Dr. LI Chun Wei, Department of Otolaryngology, National University of Singapore, National University Health System, Singapore

**Technician:** Dr. LIN Zhi Bin, Department of Otorhinolaryngology, The First Affiliated Medical Hospital of Sun Yat-Sen University, Guangzhou, China.

**PhD students:** LU Han Gui, Department of Otorhinolaryngology, The First Affiliated Medical Hospital of Sun Yat-Sen University, Guangzhou, China.

**PhD students:** CHEN De Hua, Department of Otorhinolaryngology, The First Affiliated Medical Hospital of Sun Yat-Sen University, Guangzhou, China.

**Study methods**

**Study design**

We sought to investigate the effect of SIT on the inflammatory responses in AR patients who were monosensitized to HDM. Peripheral blood mononuclear cells (PBMCs) and plasma will be isolated from AR patients before the initiation of SIT and at the end of a 2-year SIT course. Gene markers and cell populations will be detected in the PBMCs and plasma samples by using quantitative PCR, ELISA, and flow cytometry. These markers will be also evaluated in the *in vitro* allergen stimulation PBMC model. The molecular change will be correlated with the patients’ symptom scores in order to evaluate the relationship between gene levels and SIT efficacy in AR patients.

**Study subjects**

Fifty Chinese patients suffering from persistent AR with significant nasal symptoms (e.g., nasal itching, sneezing, nasal obstruction and rhinorrhea) will be recruited in the outpatient clinic in the First Affiliated Medical Hospital of Sun Yat-Sen University from Sept.8th, 2008 to Oct.28th, 2011. All AR patients are diagnosed by an ENT/allergy specialist following the criteria from the ARIA document. The inclusion criteria for the study patients are (1) age above 18 years old; (2) having persistent AR symptoms during the past two consecutive years; (3) allergic sensitization to Dermatophagoides pteronyssinus, & Dermatophagoides farina confirmed by both skin prick test (SPT) and ImmunoCap® (Phadia, Uppsala, Sweden); (4) no previous treatment of SIT; and (5) negative SPT results to other common inhalant allergens (e.g., common pollens, cockroach, fungi, and animal dander).

The serum specific IgE (sIgE) to Dermatophagoides pteronyssinus & Dermatophagoides farina was measured by the ImmunoCap test (Phadia) and a value of more than 0.35 kUA/l is considered a positive allergic response. In addition, 20 healthy volunteers (age above 18) without any allergy history are selected as a control group. The non-atopic status of healthy controls is double confirmed by a negative SPT to all common inhalant allergens and a negative result of Phadiatop test (Phadia). All recruited subjects (both AR patients and controls) do not have an infection, asthma, autoimmune diseases, or other upper airway diseases such as septal deviation, nasal polyps, or sinusitis before enrolment. Furthermore, the patients and control subjects have not received any form of steroids or SIT at least six months before the study. Both patients and healthy controls are given written consent to undergo the clinical study. The study was approved by the ethics committee of the First Affiliated Medical Hospital of Sun Yat-Sen University in Guangzhou, China.

**SIT protocol**

AR patients will receive subcutaneous SIT for 2 years. SIT is performed by ENT clinicians in the clinic, using with a standardized mite depot-allergen extract (50% Dermatophagoides pteronyssinus & 50% Dermatophagoides farina) according to the recommendations of the manufacturer Allergopharma Joachim Ganzer KG (Reinbek, Germany). The build-up phase (25 weeks) begin at 5TU/ml of the final concentration and increased to 50TU/ml, 500TU/ml, and 5000TU/ml at 5-week intervals as tolerated. Maintenance immunotherapy is administrated with the 5000TU/ml concentration bi-weekly for 54 weeks and then tri-weekly for 27 weeks. The adjustment in the dosing schedule is mainly based on the tolerance of dose and the occurrence of adverse reactions.

**Sample collection**

Two tubes (5ml each) of blood samples from AR patients are collected before the initiation of SIT (baseline, SIT-untreated) and after the end of the SIT course (the third year, SIT-treated). Control patient’s blood is also sampled at the beginning of the study. The sample collection and procession are carried in a standardized procedure: (1). Blood samples are coded confidentially, stored in ice and sent to the service lab within 3 hours; (2). One tube is for separation of plasma, and the other is for isolation of PBMC; (3). Freshly isolated PBMC is stimulated with house dust mite (HDM) extracts; (4). HDM un-stimulated and stimulated PBMC are separated into two portions, one for RNA extraction and the other for flow cytometry; (5). Plasma, and cell culture supernatants are stored in small aliquots at -80℃ until use; (6). PBMC pellets were preserved in RNAlater® (Life Technologies) at -80℃, when finishing sample collection, RNAs are isolated and the aliquots are stored at -80℃; (7). A portion of cells is fixed and is performed flow cytomtery experiment immediately. All ELISA and PCR experiments are performed at the same time in the end of sample collection. The sample process and experiments of quantitative PCR, ELISA, and flow cytometry are done by an officially registered commercial service lab (Biolink, Guangzhou, China; website, http://www.biolinkchina.com/).

***In vitro* cell stimulation**

PBMCs are cultured with condition medium containing RPMI-1640 (Sigma, St.Louis, MO), 10% heat-inactived fetal bovine serum (Sigma), and 100 U/ml penicillin and 100 μg/ml streptomycin (Invitrogen, Carlsbad, CA). PBMCs are stimulated with HDM allergen for 72h at the optimized concentration (50 μg/ml). Allergen mixture is a standardized mite depot-allergoid (50% Dermatophagoides pteronyssinus and 50% Dermatophagoides farina) (Allergopharma, Rheinbek, Germany). At the end of the stimulation, monensin (eBioscience, San Diego, CA) (2 µmol/L), PMA (eBioscience) (20 µg/L) and ionomycin (eBioscience) (1 mg/L) are added to both HDM treated and HDM untreated cultures for 5h to enhance the expression of the cytokines.

**Quantitative PCR**

Total RNA was extracted from PBMCs by using Trizol® (Invitrogen) according to the manufacture’s protocol. RNA was reversely transcribed and real-time RT PCR measurement was performed by using ABI 7300 PCR system (Applied Biosystems, Foster City, CA). Relative gene expression was analyzed using the comparative 2-ΔΔCt method.

**Cytokine assays**

Concentrations of selected cytokines in undiluted plasma from SIT-untreated and SIT-treated patients are determined by Enzyme-linked Immuno absorbent Assay (ELISA).

**Flow cytometry**

PBMCs are stained with surface markers PE-cy5-labeled anti-human CD4 monoclonal antibody (mAb) and APC-Cy7-labeled anti-human CD3 mAb at room temperature for 30 minutes. The samples are fixed with Fixation Solution (eBioscience, San Diego, CA) at 4℃for 20 minutes in dark. Cells are then re-suspended and permeabilized in Permeabilization Buffer (eBioscience) to facilitate the intracellular staining. PBMCs are stained with intracellular cytokines by using selected antibodies coated with different fluorescence dye. Samples are run on a FACSCalibur™ Flow Cytometer (BD Biosciences) and are gated by CD3/CD4 positive cells; the data are analyzed by using CellQuest software (BD Biosciences).

**VAS evaluation**

The symptom score is analyzed by VAS system according to the ARIA document. The recommended method of assessing severity of symptoms (e.g., itching, sneezing, congestion and discharge) is with the use of a VAS recorded by the patient on a 10cm line giving a score on a measurable continuum of 1 to 10. Then subjects are instructed that “Score 0” meant “overall symptoms not all bothersome” and that “Score 10” meant “overall symptoms extremely bothersome”. All patients are coded confidentially and VAS was evaluated both before and after (2 year) SIT independently by a clinician in a blinded manner.

**Statistics**

All data were analyzed using the SPSS statistical software V17.0 (SPSS Inc., Chicago, IL). The following statistics methods will be used based on different comparation: Wilcoxon matched pairs sign rank test, Mann-Whitney two-tailed test and Spearman rank analysis. Statistical significance was accepted when the p-value < 0.05.
